# Deep learning encodes robust discriminative neuroimaging representations to outperform standard machine learning

**DOI:** 10.1038/s41467-020-20655-6

**Published:** 2021-01-13

**Authors:** Anees Abrol, Zening Fu, Mustafa Salman, Rogers Silva, Yuhui Du, Sergey Plis, Vince Calhoun

**Affiliations:** 1 Tri-institutional Center for Translational Research in Neuroimaging and Data Science (TReNDS), Georgia State University, Georgia Institute of Technology, Emory University, Atlanta, GA USA; 2 School of Electrical & Computer Engineering, Georgia Institute of Technology, Atlanta, GA USA; 3 School of Computer & Information Technology, Shanxi University, Taiyuan, China

**Keywords:** Computational biology and bioinformatics, Machine learning, Neuroscience, Computational neuroscience, Learning algorithms

## Abstract

Recent critical commentaries unfavorably compare deep learning (DL) with standard machine learning (SML) approaches for brain imaging data analysis. However, their conclusions are often based on pre-engineered features depriving DL of its main advantage — representation learning. We conduct a large-scale systematic comparison profiled in multiple classification and regression tasks on structural MRI images and show the importance of representation learning for DL. Results show that if trained following prevalent DL practices, DL methods have the potential to scale particularly well and substantially improve compared to SML methods, while also presenting a lower asymptotic complexity in relative computational time, despite being more complex. We also demonstrate that DL embeddings span comprehensible task-specific projection spectra and that DL consistently localizes task-discriminative brain biomarkers. Our findings highlight the presence of nonlinearities in neuroimaging data that DL can exploit to generate superior task-discriminative representations for characterizing the human brain.

## Abstract (web summary)

Recent critical commentaries unfavorably compare deep learning (DL) with standard machine learning (SML) for brain imaging data analysis. Here, the authors show that if trained following prevalent DL practices, DL methods substantially improve compared to SML methods by encoding robust discriminative brain representations.

## Introduction

The application of machine learning for investigation of neurological and psychiatric disorders has grown greatly in the last two decades^1,2^. Standard machine learning (SML) approaches predict health-related outcomes by manipulating specific linear or nonlinear prediction functions using rules of inference. The SML methods do not learn representations but instead determine decision boundaries in the native, kernel-transformed, or feature-engineered input spaces. This is, in fact, one of the most significant limitations of SML methods in modeling brain data. An indispensable prerequisite to boosting the performance of SML approaches is reducing the dimensionality of the input space, typically enabled through hand-crafted or expert-designed feature selection (i.e., identification of a subset of variables that capture most of the information in the data) and/or feature extraction (i.e., projection of the features onto a lower-dimensional space by some linear or nonlinear data transformation) techniques^3,4^. The persistent challenge imposed by this preliminary step paved the way for the introduction of deep learning (DL) approaches. DL approaches, instead, can exploit the wealth of information available from minimally preprocessed input images to characterize the subtle patterns inherent in the input data as an integral part of the training process. The training phase of DL approaches often involve the automatic and adaptive discovery of discriminative data representations at multiple levels of hierarchy in an end-to-end (input to output) learning procedure. Application of this radically different approach in an end-to-end manner can also provision backwards mapping to the input image space through methodical interpretations, thus possibly allowing us to make inferences about brain mechanisms, for example, delineating the features in the input space that are most influential in predicting an attempted task. On the contrary, relevant spatial relationships may be lost at the dimensionality reduction stage, arguably, required for SML methods to work. DL approaches have been successful in learning more discriminative data encodings (i.e., representations) compared with their manually engineered counterparts and several automated dimensionality reduction techniques in the field of computer vision^5,6^.

DL approaches have already shown great promise in diverse applications to medical imaging data^7–10^. Several structural and functional brain imaging modalities are now being actively used to study mental health non-invasively. Although SML approaches have contributed to these efforts making significant advances^11–16^, the relatively newer whole-brain DL approaches are just beginning to record successes, particularly in the image preprocessing, diagnostic classification, regression, disease characterization, and disease prediction domains^17–24^. Concurrently, similar to preceding influential technologies, expectations of the future performance of DL frameworks sometimes grow out of proportion to reality. For example, their viability to learn subtle properties of complex multiscale brain imaging data and potential to scale may be hyped^25^. Perhaps as a reaction to this inflation, recent critical commentaries unfavorably compare DL with SML approaches^26–28^. Yet, these commentaries are limited in a few fundamental ways, and their conclusions must be considered at specific merits as reviewed next.

First, although there is a wealth of information to be learned from manually designed or automatically pre-engineered structural or functional brain imaging features, these do not necessarily comprise the most idealistic choices for training and exhaustively benchmarking DL methods. This is attributable to the fact that the use of pre-engineered features deprives DL of its main advantage: representation learning from raw or minimally preprocessed input space. Among these critical studies, Schulz et al.^28^ compare SML and DL methods on several tasks including a combined age and gender task and separate age regression and gender classification tasks. Their study^28^ focuses on using several pre-engineered features and 2D representation learning on partial data (i.e., central brain slices), whereas additionally testing 3D representation learning on whole-brain gray matter voxels, reporting comparable performance for SML and DL for all analyses. Similar to the use of pre-engineered features, learning from central 2D slices conveys less information than would learning from all 2D slices, or perhaps whole-brain 3D images. Furthermore, for 3D whole-brain learning, this study^28^ attempts separate age regression and gender classification tasks on whole-brain 3D images by replicating the DL model and training pipeline in Peng et al.^29^. Despite using the same DL model, training pipeline, and data set, these two studies outline contradictory results for the age regression task on whole-brain images; with Peng et al. reporting a significantly higher performance for DL (as compared to SML), whereas Schulz et al. report no difference in performance in this task, thus demanding further confirmation for reproducible research. Incidentally, we explain this discrepancy by additionally extensively testing the Peng et al. model and pipeline, and demonstrating that this stark difference can be explained by a mere miscalculation (coding bug) in Schulz et al. In addition, He et al.^26^ and Thomas et al.^27^ both report no significant improvement in performance with DL from their expansive experimentation on static functional connectivity and several temporal rest-fMRI features, respectively. The voxel-wise or network-level fMRI features are highly informative. Nevertheless, for an exhaustive comparison, it is essential to develop and test DL models that can learn directly from 4D data, allowing learning representations in the input fMRI data space in an end-to-end manner. Hence, the experiments conducted in these two fMRI studies are limited to training DL models on pre-engineered features similar to the majority of experiments in Schulz et al., thus not allowing exhaustive benchmarking; however, the claims made in these two studies are rather balanced as their conclusions are presented within the context of the experiments performed.

In this work, we correct for the above-mentioned deficiencies of prior benchmarks in a principled, comparative analysis of structural brain imaging data and show how vital the representation learning part of DL is for its performance. To this end, we systematically profile the classification performance and empirical time complexity of several SML and DL methods on a 10-way age and gender-based classification task^28^ using a large data set of structural magnetic resonance imaging (sMRI) images. We conduct this comparison for a range of training sample sizes to compare and contrast the asymptotic behavior in performance improvement and relative time complexity of the two approaches. In addition, we probe the consistency in the task-discriminative power of the DL embeddings by evaluating the performance of SML methods trained on these features. We also validate the observed performance trends on additional tasks, including a gender classification task, an age regression task and a mini-mental state examination (MMSE) regression task. Furthermore, to confirm the reproducibility of our results, we conduct an extensive comparison of our DL models and training pipeline with that proposed in recent research^29^. Finally, we conduct post hoc analyses to assess the degree of consistency and robustness of the validated task-specific DL models and probe the rationality of task-specific brain regions via model introspection.

The choices for evaluating our key objectives with the tasks undertaken in this work were driven by the high scientific utility of learning age-related and gender-related correlates of MRI and the need to evaluate performance on an independent cognitive task in a patient population. In particular, age prediction is a popular proxy benchmark for determining other scientifically exciting questions such as cognitive function, mental disorders, etc.^30–38^. Similarly, gender may also significantly impact cognitive function, including memory, emotion, perception, and more, and is widely studied in this regard^39–43^. Moreover, methodological comparison of models based on these discriminative variables makes much more sense and brings much more value when done using models with a high discriminative performance rather than on models that are trained to random performance. Finally, although our study results show the best performing approach for fitting a discriminative classifier or regressor for the attempted sample tasks and brain imaging modality, a similar analysis could be extended to other inference tasks and modalities. Next, we present the findings of our exploratory work.

## Results

### DL capacitates more discriminative features

**Fig. 1:**
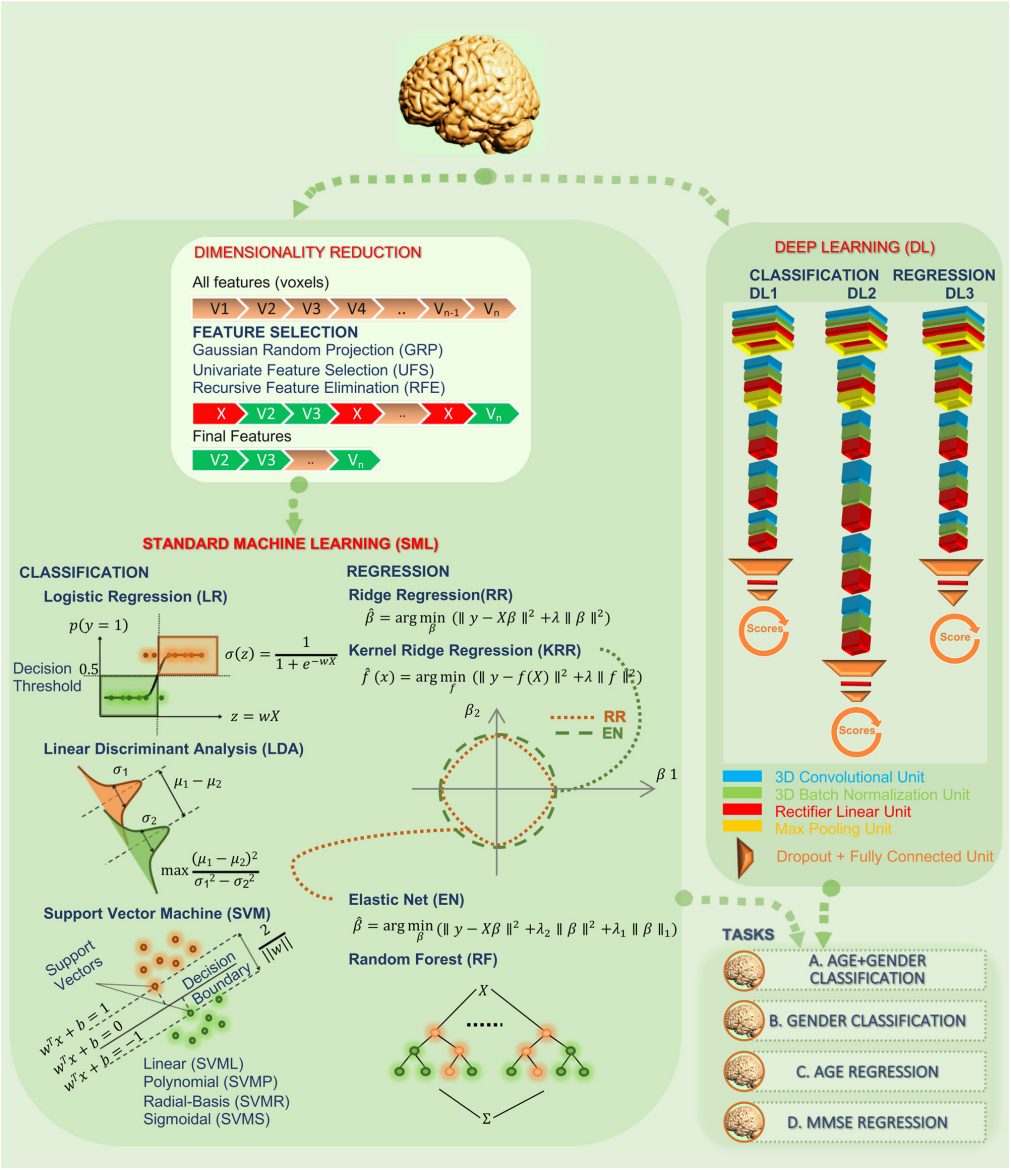
Systematic comparison of classification and regression performance of SML and DL methods. Performance for several SML and DL methods for multiple classification and regression tasks was assessed using UK Biobank structural MRI data of 12,314 subjects (tasks A, B, and C) and ADNI structural MRI data of 828 subjects (task D). A rigorous cross-validation procedure was conducted via 20 random partitions of the data into training, validation, and test sets. For each repeat, the hyperparameters were tuned on the validation data and reported performance was evaluated on the held-out test set. The SML models were trained/tested on gray matter features reduced by three different feature reduction methods. In contrast, the DL models were trained on the unreduced (i.e., 3D voxel space) preprocessed gray matter maps.

We systematically evaluated how the performance (as measured by accuracy and run time) of the SML and DL models scaled as a function of training sample size in a 10 class age and gender classification task (i.e., five age groups from each gender) evaluated in a standard repeated (*n* = 20), stratified cross-validation (CV) procedure as outlined in Fig. 1. We used gray matter volume maps extracted from the sMRI data of 12,314 unaffected (i.e., with no diagnosed or self-reported mental illness) subjects for this assessment. To establish a performance baseline for the SML methods, we included three linear SML models—linear discriminant analysis (LDA), logistic regression (LR) and support vector machine with a linear kernel (SVML) and three nonlinear SML models—support vector machines with a polynomial (SVMP), radial-basis function (SVMR), and sigmoidal (SVMS) kernels (similar to Schulz et al.^28^). In addition, we tested two nonlinear DL models—both 3D CNN variants of the AlexNet architecture^44^ that differed primarily in the network depth (depth_DL2_ > depth_DL1_) and the number of channels in the convolutional layers. Given that feature extraction is an indispensable measure of boosting performance of linear and kernel-based SML methods, we reduced the gray matter maps with three dimensionality reduction methods: Gaussian Random Projection (GRP), Recursive Feature Elimination (RFE) and Univariate Feature Selection (UFS) as detailed in the methods section. We trained the DL architectures directly on the unreduced input space of 3D gray matter maps to fully utilize their representational power.

**Fig. 2:**
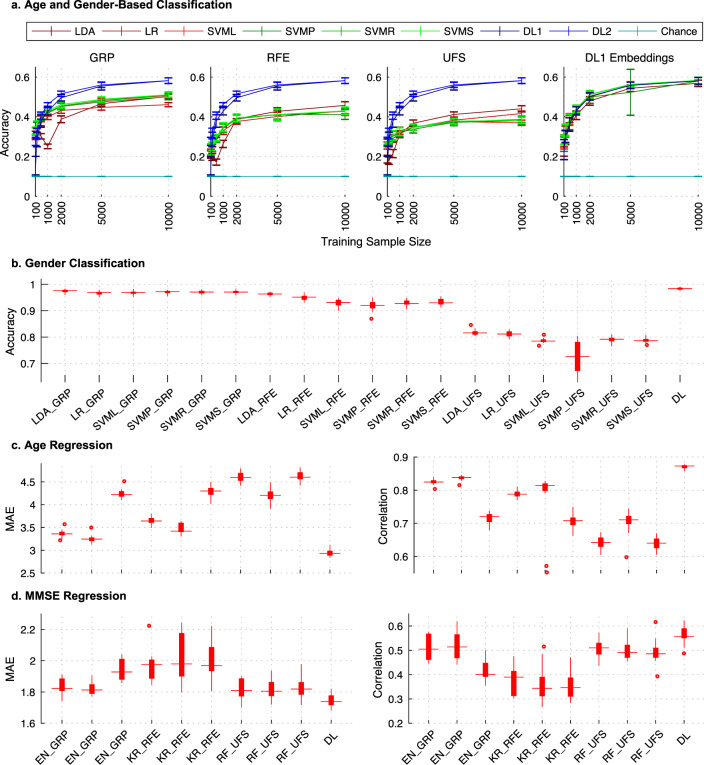
DL capacitates more discriminative features across multiple classification and regression tasks. **a** Age and gender-based classification task. We test six standard machine learning (SML) methods, including linear (red hues—*LDA* linear discriminant analysis, *LR* logistic regression, *SVML* support vector machine with a linear kernel) and nonlinear models (green hues—SVMP, SVMR, and SVMS, which are abbreviations for SVM models with a Polynomial, Radial-Basis Function, and Sigmoidal kernel, respectively) by reducing the high-dimensional whole-brain gray matter by three dimensionality reduction (DR) methods (*GRP* gaussian random projection, *RFE* recursive feature elimination and *UFS* univariate feature selection) and compared against two deep learning (DL) models (blue hues: DL1 and DL2) trained on 3D whole-brain gray matter. This task was performed with a repeated (*n* = 20) random sub-sampling cross-validation scheme on UK Biobank MRI data (*n* = 12,314; *n*_validation_ = 1157; *n*_test_ = 1157) on a range of training sample sizes that varied between 100 and 10,000 samples. Both DL classification models significantly outperformed (evaluated using a two-tailed paired sample *t* test) all six SML classification models regardless of the DR method for all training sizes beyond the test sample size. In addition, superior feature extraction of the DL models was immediately evident as the SML models trained on the DL1 representations (DL1 Embeddings panel on top right) performed equally well. The error-bars highlight the mean values ±1 SE across the 20 cross-validation repetitions, whereas the horizontal line along the normalized accuracy level of 0.1 represents the chance probability for this 10-way classification task. **b** Gender classification task. The largest sample (*n*_train_ = 10,000; *n*_validation_ = 1157; *n*_test_ = 1157) was evaluated for gender classification using the same cross-validation procedure. For this task, the tested DL model significantly outperformed all six SML classification models for all DR methods. The label abbreviations on the *x* axis of this plot (except DL) refer to this task performed by implementing a combination of the listed SML model and the DR method, whereas DL represents this task performed with our DL1 model on 3D whole-brain gray matter. **c** Age regression and **d** Mini-mental state examination (MMSE) regression tasks. We evaluate three SML methods, including the elastic net (EN), Kernel Ridge (KR), and Random Forrest (RF) regression methods on features estimated by all three DR methods. The label abbreviations on the *x* axis of this plot (except DL) refer to these tasks performed by implementing a combination of the listed SML model and the DR method, whereas DL represents this task performed with our deep vanilla regressor model (DL3) on 3D whole-brain gray matter. The age regression task was evaluated on the largest training sample size (*n*_train_ = 10,000; *n*_validation_ = 1157; *n*_test_ = 1157), whereas the MMSE regression task was implemented on ADNI MRI data (*n* = 828; *n*_train_ = 428; *n*_validation_ = 200; *n*_test_ = 200) using the same cross-validation procedure. The mean absolute error (MAE) and the Pearson correlation coefficient (PCC) (between the true and predicted values) performance metrics were estimated to compare performance for the DL vanilla regressor model and the three SML regression models for both regression tasks. Both tasks reported statistically significant improvement in MAE (a decrease) and PCC (an increase) for the DL regressor as compared to the SML regression models as evaluated using a two-tailed paired sample *t* test. For the boxplots plotted in **b**, **c**, and **d** panels, the box shows the inter quartile range (IQR between Q1 and Q3) of the data set, the central mark (horizontal line) shows the median and the whiskers correspond to the rest of the distribution based on IQR [Q1-1.5*IQR, Q3 + 1.5*IQR]. Beyond the whiskers, data are considered outliers and represented by red circles. Source data are provided as a Source Data file.

We found that the two DL models significantly outperformed the SML models evaluated on each of the reduced feature spaces (Fig. 2a). For the highest sample size (*n* = 10,000), the DL models reported 10 class classification accuracies of 58.19% (DL1) and 58.22% (DL2), respectively (note, this task had a chance probability of 10%). In contrast, the SVMS and LDA models reported the highest accuracies for the GRP (SVMS: 51.15%), RFE (LDA: 45.77%), and UFS (LDA: 44.07%) features. Indeed, the GRP method resulted in the most discriminative features for all SML models, followed by the RFE method. Although both DL models consistently reported significant improvement (*p* = 1.03 × 10^−14^ for a two-tailed paired sample *t* test comparing DL1 and best performing SML model) with an increase in training sample size, this observation was not necessarily true for the SML models. For example, the performance of LDA on GRP features dropped initially, possibly owing to a smaller training sample size than the validation and test data sizes. In addition, as expected for sparse models, no significant improvement was observed in the performance of the SVMP for the RFE features, and SVML and SVMS for UFS features with an increase in training sample size from *n* = 5000 to *n* = 10,000. Interestingly, our SML baseline is also considerably higher than the SML baseline in Schulz et al. at the same training sample sizes. This observation could be owing to (1) differences in the parameter for the maximum number of iterations used by the SML solvers for convergence (we allowed 10,000 iterations for convergence as compared to 100 or 1000 in the comparative work, thus increasing the probability of convergence in our runs), (2) preprocessing differences (for example, smoothing kernel size, gray matter masks and input data—namely raw versus modulated gray matter probability images), and (3) validation/test data sizes (for example, 1157 in our study versus 650 in the comparative work, thus giving our SML models an additional advantage of generalizing better) but these differences remain to be confirmed. Despite this improvement in the SML baseline, our results show that DL significantly outperforms SML on this task.

Interestingly, the performance improvement for DL models showed asymptotic behavior similar to SML methods, though with significantly higher performance. That is, for the DL methods as well, the performance gains are slowing down, although the models do continue to improve. Whether the slowdown is impactful and where is the point of diminishing returns occurs is application dependent. Our observation demands a further confirmation as there are many ways to potentially score further gains in performance by testing even deeper models, finetuning the existing DL models, and exploring other DL approaches. Furthermore, if the DL models are indeed extracting superior (i.e., more discriminative) features consistently, the lower-dimensional encodings generated by them should result in significantly improved performance if used as input features of the SML models as compared with the three tested dimensionality reduction methods. To ascertain this, we conducted a post hoc analysis where we evaluated the performance of the SML models on the trained encodings from the DL1 model (i.e., the output of the first fully connected layer in DL1). As expected, we observed a significant increase in the performance of the SML methods applied to test data, providing evidence that the SML methods could perform equally well if using the DL encoded feature spaces (Fig. 2a).

We performed additional comparative analyses on a few classification and regression tasks to further validate the comparative performance of SML and DL methods in learning from the brain imaging data. For the gender classification task (Fig. 2b), the DL1 classification model reported a mean classification accuracy of 98.34% for the largest training sample size (*n* = 10,000), a significant improvement (*p* = 4.69 × 10^−7^; two-tailed paired sample *t* test) over the best performing SML classification method (i.e., the LDA method for GRP features that reported an accuracy of 97.45%). For the two regression tasks, we developed and tested a deep vanilla regression framework (DL3) based on the DL1 classification model to predict age and MMSE scores. In these regression tasks, we compared the performance of our DL regression model with that of SML regression methods including elastic net (EN), kernel ridge regression (KRR) and random forest (RF) ensemble learning. To quantify the performance in these two regression tasks, we relied on two performance metrics: (1) the mean absolute error (MAE) between the true and predicted values of interest and (2) the Pearson correlation coefficient (PCC) between these values. Our results on the age regression task (Fig. 2b) reported a significantly lower MAE (mean MAE_Age_ = 2.94; *p* = 2.76 × 10^−11^; two-tailed paired sample *t* test) and significantly higher PCC between predicted and true age for the DL models (mean PCC_Age_ = 0.87; *p* = 2.59 × 10^−12^; two-tailed paired sample *t* test) as compared with the best performing SML regression method (i.e., the KRR method for GRP features; mean MAE_Age_ = 3.25; mean PCC_Age_ = 0.84). Likewise, our results on the MMSE regression task (Fig. 2c) reported a significantly lower MAE (mean MAE_MMSE_ = 1.74; *p* = 2.65 × 10^−3^; two-tailed paired sample *t* test) and significantly higher PCC between predicted and true MMSE values for the DL models (mean PCC_MMSE_ = 0.56; *p* = 2.99 × 10^−3^; two-tailed paired sample *t* test) as compared with the best performing SML regression method (i.e., the KRR method for GRP features; mean MAE_MMSE_ = 1.81; mean PCC_MMSE_ = 0.51). Notably, our results on regression tasks demonstrate that representation learning in DL models may be instrumental in learning more precise continuous scales in neuroimaging clinical data (i.e., regression tasks) in addition to making superior categorical predictions (i.e., classification tasks).

### DL presents lower empirical asymptotic complexity in relative computational time

The theoretical and empirical computational time complexities of machine learning algorithms are not critical considerations for current clinical applications since inference is commonly applied at the patient level. Instead, we compare the computational time complexities of standard implementations of the SML and DL methods to address the reactionary, but inconsistent response that standard implementations of DL methods have a high computational time complexity and take forever to run. In contrast, the high order of computational complexity growth of the standard, CPU-based SML implementations on large training data sets is often overlooked. Indeed, this demonstration is crucial at the current stage in the neuroimaging community, as researchers may be discouraged from undertaking the use of DL methods based on this reactionary, but inaccurate response.

A theoretical computational time complexity analysis is generally sufficient to describe algorithms’ asymptotic behavior and can be more informative than an empirical one. However, it is not straightforward to derive the theoretical computational complexity for ML algorithms owing to the several, highly variable steps involved in their training routines. The algorithms used by SML as well as DL methods are parameter and implementation-dependent (e.g., optimization solver, caching, shrinking, parallelization, and model compression), and their standard implementations may be heterogeneous owing to external algorithmic calls. This situation is further exacerbated with iterative training and the need for hyperparameter tuning (for example, grid search with CV), thus requiring the algorithms to run several times within the same experiment (again, with multiple adaptive/variable parameters). Owing to the above constraints, comprehensive analyses of theoretical time complexity of the machine learning algorithms are sparingly and generally non-exhaustively assessed in previous computer science literature, although empirical comparisons are commonplace^45–53^.

**Fig. 3:**
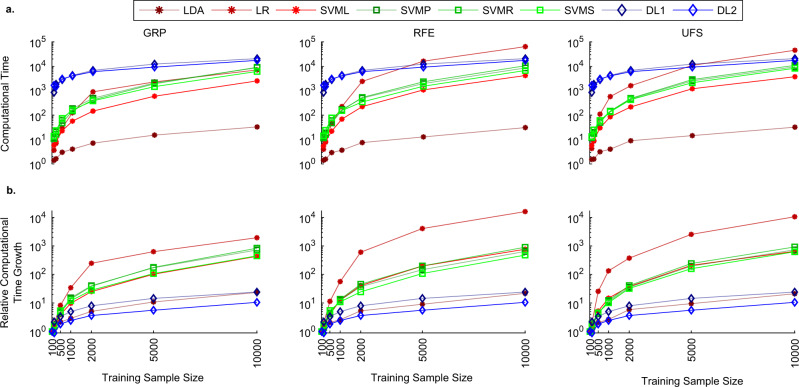
Systematic comparison of computational time complexity. This analysis compared the growth in computational time with an increase in the training sample size across all models and dimension reduction methods in the age and gender-based classification task. **a** The DL models recorded higher run times but showed a more linearly increasing trend with an increase in training sample size. On the other hand, the SML models presented a quadratic trend (except for the LDA method). As a result, the difference between the recorded run times between the two classes of models decreased as the training sample size increased. **b** To further validate this trend, we conducted a similar analysis on a metric for relative computational time growth (defined as the computational time for the given training sample size normalized by this metric for the smallest sample size). This analysis suggested a lower asymptotic complexity in the relative run time growth for DL models. Source data are provided as a Source Data file.

Hence, we pursued empirical evidence to determine the growth in the computational time of the two classes of methods as a function of training sample size in the age and gender-based classification task. Figure 3a presents the average computation time of all tested models. This comparison illuminates a higher growth rate of computation time for most of the SML models, as the recorded differences for the two classes of models diminished with increasing training sample sizes for all SML models except LDA. Furthermore, to confirm if this observation indeed implied lower empirical asymptotic complexity for DL models, we estimated a relative computational growth rate metric by normalizing the computation time with the computation time for the smallest training sample size. The results of this analysis (Fig. 3b) are an empirical evidence of lower growth rate in computational complexity of DL models compared with all SML models except LDA.

Furthermore, we note that the computation time of the SML models did not include the time used for the dimensionality reduction. In addition, the GPU implementation for DL models used the same number of CPU threads (*n* = 8) as that for the SML models. As the relative computational time metric was estimated relative to a baseline (i.e., smallest sample size for the same method), we speculate the difference in nature of the implementations for the two classes of methods to not result in a significant change in the relative metric, even though the non-normalized metric in Fig. 3a could be expected to drop for the SML models if a GPU- based implementation were used. Critically, our focus on comparing the computational growth rate for the commonly used modern implementations of these models assumes high significance as bigger data samples are becoming increasingly available for research and can be safely expected to grow even bigger in coming years.

### DL learns meaningful brain representations that span a comprehensible projection spectrum

**Fig. 4:**
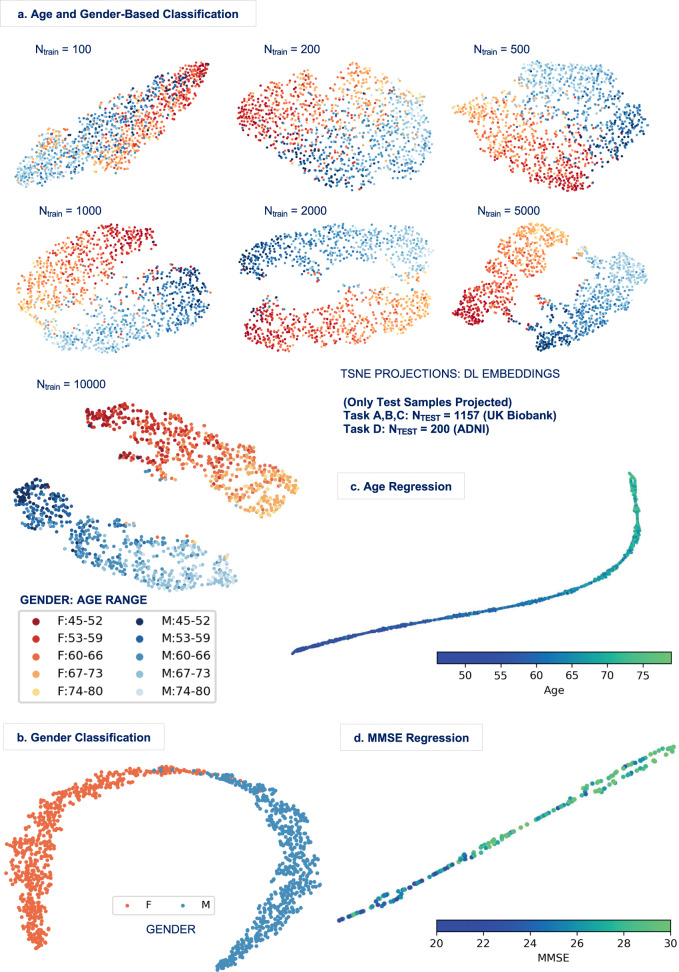
Projections of the embeddings from the validated DL models. **a** The projections of the embeddings inferred for the combined age and gender task were compared across a range of training sample sizes. Representational patterns of the brain are indeed learnt. In fact, they distill continually with increasing training sample size, and eventually evolve into separate gender clusters (i.e., red/F/female and blue/M/male clusters), both presenting a gradual spectrum of age (i.e., traceable light colored to dark colored). **b** The gender classification task (*F* females, *M* males) also revealed distinct clusters with very few outliers. **c**, **d** The projections for the age and MMSE regression tasks also revealed comprehensive trends in the spectrum, thus confirming that the implemented DL methods were indeed able to learn the task-specific brain representations.

If the DL methods are indeed learning embeddings that represent the brain in low dimensional space, the encodings in deeper layers (further from the input) must be discriminative for the attempted task. Thus, for the undertaken age and gender-based classification task in this work, we can expect the inferred DL encodings to capture meaningful age and gender information from the high-dimensional input data. Furthermore, we can anticipate such information in the captured patterns to continually distill with an increase in training sample size. To validate this statement, we conducted a post hoc analysis by projecting the learnt DL1 embeddings (i.e., the output of the first fully connected layer in the DL1 architecture) onto a two-dimensional space using t-distributed stochastic neighbor embedding (t-SNE)^54^ for the entire range of training sample sizes, and color-coding the two-dimensional projection spectrum by the class labels. The t-SNE algorithm works on placing two-dimensional representations maximally preserving their distances in the original space; thus, if the embeddings contain pronounced age and gender information, subjects of the same gender and similar age are expected to end up nearby. The t-SNE layouts of the learnt DL representations in Fig. 4a reveal meaningful refinement of the learnt patterns with increasing training sample size, with the progressive evolution of an explicit bi-modal structure (i.e., formation of two distinct gender clusters) both modes of which manifest a comprehensible, gradual spectrum of age. More specifically, we can see separate gender clusters ordered in increasing age from one end of the spectrum to the other, although some outlier observations do exist.

The nonlinear projections of the inferred embeddings in the other three learning tasks also showed comprehensive trends, which further verified the rationality of the learning and inference processes in the DL methods. The gender classification task revealed distinct clusters with very few outliers (Fig. 4b). In addition, continuous gradual spectra with increasing age (Fig. 4c) and MMSE (Fig. 4d) values from one end of the spectrum to the other were observed for the undertaken age and MMSE regression tasks, respectively. Hence, we can conclude that the implemented methods were indeed able to learn the task-specific representational patterns of interest from brain imaging data.

### DL enables robust relevance estimates for human brain regions

A critical dimension for validating the robustness of an algorithm is the similarity in the predictions across its independent repetitions. Hence, we sought to determine whether the validated DL models estimated prediction relevance of the brain regions in the classification decisions in a consistent pattern across their independent runs. For this, we recorded the saliency maps for the multiple repetitions (*n* = 20) that varied in the randomly sampled training, validation and testing data for the highest training size (*n* = 10,000) in the age and gender-based classification task. The saliency maps were estimated through two standard approaches, namely gradient-based backpropagation^55^ (GBP) and network occlusion sensitivity analysis^56^ (NOSA). The GBP approach computes the gradient of the class score with respect to the input image to determine the relevance of each pixel with respect to the classification decision. In the NOSA method, brain functional networks are occluded one at a time, their class probabilities are re-evaluated, and their relevance in the classification decisions is estimated proportional to the reduction in target class probability. Both of these methods are sensitive to the data and network architecture^57^, thus perfectly fitting in the scope of the attempted classification task in this work.

**Fig. 5:**
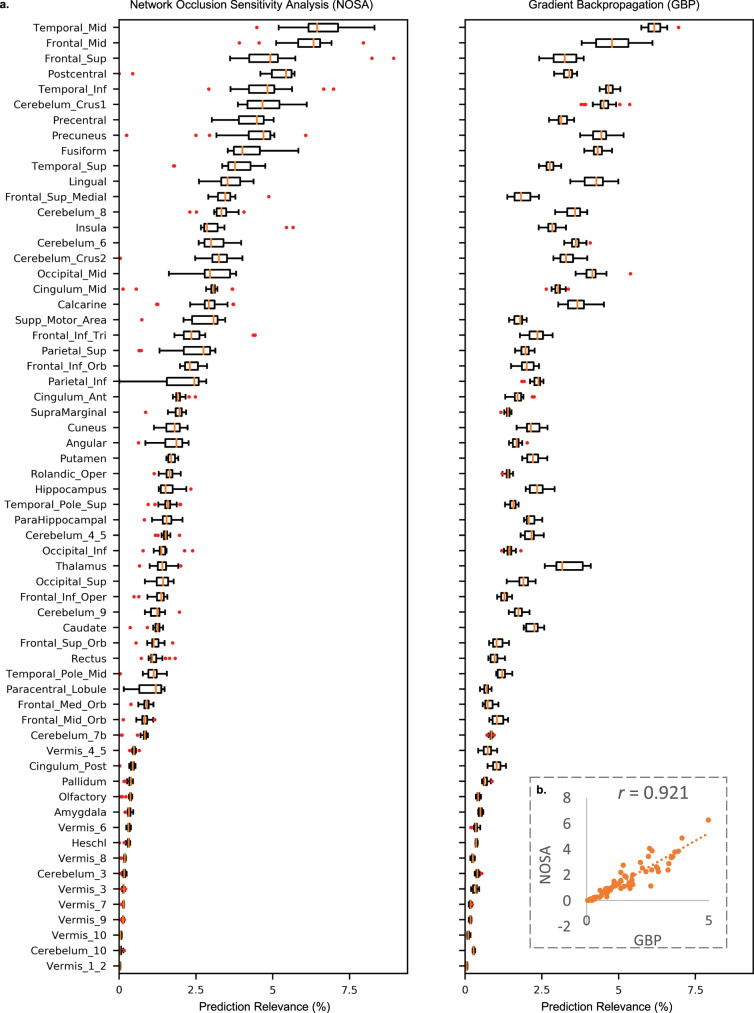
Task-discriminative relevance estimates for the AAL atlas for the network occlusion sensitivity analysis (NOSA) and gradient backpropagation (GBP) approaches for the age and gender-based classification task. **a** Aggregate relevance of each brain region was estimated for each independent (*n* = 20) cross-validation repetition for the largest training sample size runs (*n*_train_ = 10,000, *n*_validation_ = 1157 and *n*_test_ = 1157 subjects). Thus, for both saliency methods, each boxplot outlines the variation in the mean relevance percentages for each corresponding brain region across the independent (*n* = 20) cross-validation runs of the classification task. In these boxplots, the box shows the inter quartile range (IQR between Q1 and Q3) of the data set, the central mark (horizontal line) shows the median and the whiskers correspond to the rest of the distribution based on the IQR [Q1–1.5*IQR, Q3+1.5*IQR]. Beyond the whiskers, data are considered outliers and represented by red circles. These estimates generally spanned a narrow range (except for few outliers runs for some brain regions), and a comparison of these standard approaches confirmed consistency in the trends for most of the brain regions. Note, the AAL atlas brain regions are sorted from higher relevance to lower relevance for the NOSA approach for this illustration. **b** The NOSA and GBP saliency mapping methods showed a high correlation value (*r*) of 0.92 in the mean relevance estimates for each brain region (listed on the *y* axis of **a**), thus confirming the consistency in the relevance obtained from the learned DL representations. The dotted line represents the least squares fit for this relationship. Source data are provided as a Source Data file.

Figure 5a presents the task-discriminative relevance percentages based on the highest sample size computed for these approaches on the automated anatomical labeling (AAL) brain atlas^58^. Despite some variation in the ranking orders of the merged brain networks, both saliency approaches estimated similar prediction levels for most of the brain networks. The mean relevance estimates for the AAL brain atlas for both approaches and scatterplot of these metrics comparing the two approaches (*r* = 0.921) are illustrated in Fig. 5b. Overall, these initial results clearly suggest robustness in the relevance estimates and thus the high potential of the undertaken DL approach to record consistent representations of the brain imaging data. In view of such positive evidence, future DL applied to brain imaging data should investigate incorporate saliency mapping into learning formulations more comprehensively.

### DL reveals rational task-specific relevance distributions of discriminative biomarkers

**Fig. 6:**
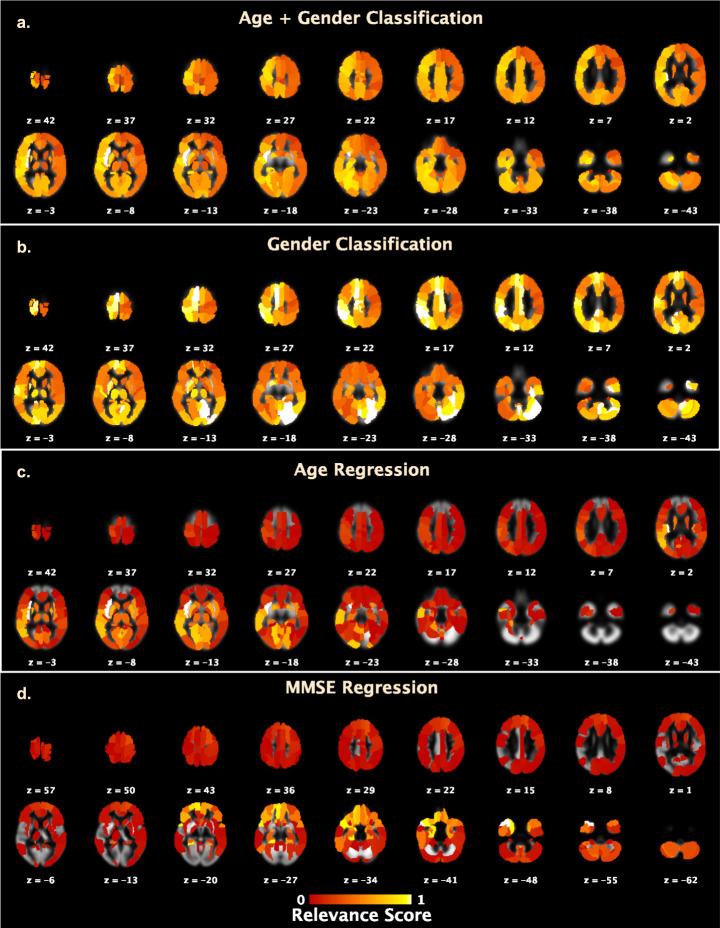
Visualization of task-specific distributions of discriminative biomarkers. The rationality of the DL model decisions was verified by examining the peak activations in the task-specific aggregate saliency maps. The panels above present the axial slices of the aggregate saliency maps for each of the undertaken classification/regression tasks - **a** age and gender-based classification, **b** gender classification, **c** age regression and **d** mini-mental state examination (MMSE) regression. Brain regions determined as the most discriminative of the undertaken tasks by the validated DL models showed a high general correspondence with previous reports in neuroimaging literature.

Here, we perform DL model introspection to qualitatively assess the brain regions most discriminative of each undertaken learning task and discuss the relationship between these revelations and previous findings in the literature. For this assessment, we estimated the task-specific distributions by averaging the aggregate saliency maps across the different CV repetitions, normalizing the averaged aggregate maps to the [0,1] range and smoothing this data for each of the undertaken tasks. As illustrated in Fig. 6a, the aggregate saliency maps for the combined age and gender classification task featured regions distributed in (1) the central structures of insular cortex and putamen, middle, and anterior cingulate gyrus, (2) the temporal lobe involving hippocampus, parahippocampal gyrus, amygdala, fusiform gyrus, Heschl’s gyrus and inferior/middle/superior temporal gyrus, (3) the occipital lobe involving the calcarine fissure, cuneus, lingual gyrus, inferior/middle/superior occipital gyrus and angular gyrus, (4) the frontal lobe including the Rolandic operculum, frontal superior and middle gyrus, the orbital and opercular inferior frontal gyrus and precentral gyrus, (5) the parietal lobe involving the precuneus gyrus, supramarginal gyrus, postcentral gyrus, and inferior parietal gyrus, and (6) the cerebellum (4/5/6/8/Crus1). As we will see next, these activations showed a high degree of correspondence with previously reported patterns of gender-related (Fig. 6b) and age-related (Fig. 6c) gray matter differences. Further investigation into this combined task can be meaningful, for example, to analyze gender-specific differences in aging, but we defer a systematic study of those effects to a dedicated work and instead review the rationality of our findings for the separate gender and age tasks next.

Our findings from the gender classification task, as illustrated in Fig. 6b, are consistent with previous studies investigating regional sex dimorphism within the brain, with highest aggregate saliency reported in the left parietal inferior lobe^59^, and other parietal lobe regions^60,61^ distributed over the angular gyrus, precuneus, superior parietal gyrus, and postcentral gyrus. The most salient activations for this task also spanned the occipital regions including the right inferior occipital lobe^62^ and cuneus, followed by the calcarine, lingual gyrus, and middle/superior occipital lobe regions^60^. Other key gender-discriminative activations were distributed in the central structures of thalamus and putamen^60,61^, posterior/middle/anterior cingulate gyrus^61,63–65^, the fusiform gyrus^60^, and Heschl’s gyrus regions^66^ and several cerebellum (6/8/9/Crus1 and Vermis 6/7/8/9) regions^61^. Our results also corroborate and extend significant relevance of the medial surface of superior frontal gyrus and paracentral lobule in this task, as also reported previously^60,65^.

For the age regression task, highest aggregate saliency was reported in the insular cortex and subcortical structures including putamen, thalamus, pallidum, and caudate nucleus (Fig. 6c), with patterns consistent to those that have been extensively reported^67–71^. Ventricular enlargement occurs in normal aging^72^, which we speculate might be correlated to the observation of enhanced activations in these subcortical gray matter regions on the periphery of the ventricles. Next, in agreement with previous brain aging reports, the most discriminative brain regions were additionally distributed through the occipital lobe including the lingual gyrus, calcarine, and the inferior/middle/superior occipital lobe AAL atlas regions^73^. Our findings also confirm significant relevance of the hippocampus and amygdala in the medial temporal lobe, the superior/middle temporal lobe regions^71,74–76^, and inferior frontal gyrus and rolandic operculum frontal regions^70,74^ in learning generalized patterns of age-related gray matter changes. Additionally, high saliency levels were also distributed in the supramarginal gyrus, and several cerebellum (4/5 and vermis 3/4/5/6) regions^77^ for the age regression task.

At lastly, we discuss the discriminative brain regions identified by the validated DL models in the MMSE regression task (Fig. 6d) in relation to previous work that has linked cognitive changes in old age to specific structural abnormalities in the brain. Consistent with a recent study^78^ directly investigating neuroanatomical correlates of MMSE in mild cognitive impairments (MCI)/AD and other MCI/AD works in literature, the most salient regions for this task were distributed mainly in the temporal lobe^79–84^ and prefrontal lobe^85–88^ regions. Specifically, the most salient principal medial temporal lobe activations included the hippocampus, parahippocampal gyrus, and amygdala regions, whereas other temporal lobe activations were localized in the middle and superior temporal poles, fusiform gyrus, and the temporal middle and inferior gyrus regions. In addition, the key prefrontal lobe activations were spread throughout the orbitofrontal cortex (inferior/middle/superior and medial regions) and in some regions in the middle and superior frontal gyrus, olfactory cortex, and gyrus rectus. Overall, our findings from the undertaken classification and regression tasks suggest that DL methods reveal rational task-specific distributions of discriminative biomarkers. Given such informative evidence, future approaches applying DL to brain imaging data should investigate incorporating saliency mapping into learning formulations more comprehensively. Supplementary Video 1 shows an animation across the different brain slices for all tasks.

### Comparative analysis confirms reproducible DL on brain imaging data

In this section, we assess the reproducibility of our DL results by extensively comparing our DL model and training pipeline to the simple, fully convolutional network (SFCN) DL model and training pipeline proposed by Peng et al. and replicated by Schulz et al. For this, we compare our pipeline (DL models, training pipeline and our custom training code referred to as DL Abrol^@^) with other pipelines referred to as SFCN Abrol^@^ (SFCN DL model, our training pipeline and our custom training code), SFCN Schulz^@^ (SFCN DL model, Schulz et al. training pipeline and our custom training code) and SFCN Schulz^*^ (SFCN DL model, Schulz et al. training pipeline and computer code). In this analysis, we observed that the contradicting results of Schulz et al. as evaluated with the SFCN Schulz^*^ pipeline are owing to a coding bug in batch-wise performance evaluation method of their 3D CNN. Specifically, for their 3D CNN analyses, the true and predicted target arguments are swapped in the loss method (lines 89–91, https://github.com/maschulz/deeperbrain/blob/bdb262833c33a8ef714ad38b252c8ec0bd6dca36/subanalyses/3d/base.py) while evaluating the sklearn R2, MAE, MSE, and accuracy methods. The *R*^2^ metric is the ratio of explained sum of squares (sum of squares of differences in predicted values and the actual sample mean) and the total sum of squares (sum of squares of differences in the actual values and the actual sample mean), and therefore sensitive in the order of predicted and actual targets. They only report the coefficient of determination (*R*^2^) for the age regression task and accuracy for the classification task; hence, the lack of reporting a directly comparable metric (i.e., MAE) may have limited Schulz et al.’s ability to detect or resolve this miscalculation. Similarly, another inconsistency is the lack of reporting the 3D CNN analysis for the combined age and gender classification task, the central task of their work. Regardless, owing to the bug, the R2 metric is estimated incorrectly, whereas other metric values (for example, MAE, MSE, and accuracy) remain unchanged by design (for example, a permutation of variables does not affect absolute values or squared values of their differences). We correct for this miscalculation in the SFCN Schulz_C^*^ pipeline (SFCN DL model, Schulz et al. training pipeline and corrected computer code). In addition, since the reported bug does not affect the accuracy metric, SFCN Schulz* and SFCN Schulz_C* pipelines are equivalent in the classification tasks and hence considered only once (as SFCN Schulz*) for the classification tasks. We evaluate all of these pipelines on the same data partitions as in all previous analyses.

**Fig. 7:**
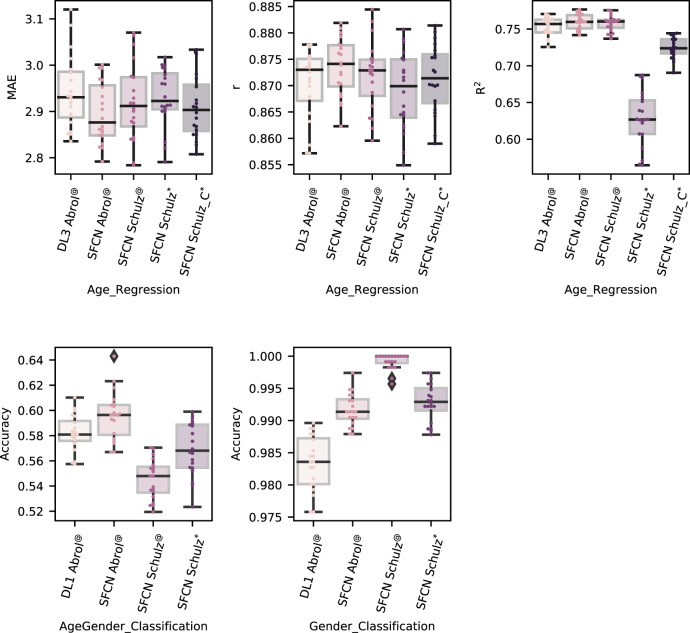
Comparative analyses confirming reproducible DL research on brain imaging data. A comparative analysis with the simple fully convolutional network (SFCN) DL model and training pipeline used in Peng et al. (2020) and Schulz et al. (2020) confirmed similar performance levels as our DL models and training pipeline, thus providing a great insight into the reproducibility of these brain imaging research objectives with DL. Several pipelines that differed in the combination of the used DL model (“DL1”, “DL3”, or “SFCN”), training settings (for example, choice of optimizer, learning rate, early stopping parametrization, etc. highlighted as “Abrol” if using similar to our work, and as “Schulz” or “Schulz_C” if using similar to comparative work) and training code (labeled with the “@” superscript when implemented with our custom training code and the “*” symbol when using the PyTorch lightning trainer as in Schulz et al.). Note, “Schulz_C” denotes the case when the coding bug in this comparative work was corrected. Specifically, the distributions of the mean absolute error (MAE), Pearson correlation coefficient (*r*), and coefficient of determination (*R*^2^) metrics are compared for the age regression task (top row), and the distribution of classification accuracy is compared for the two classification tasks (bottom row). Each boxplot shows the discriminative performance on unseen test data for the cross-validation repetitions (*n* = 20) for the highest training sample size (*n*_train_ = 10,000; *n*_validation_ = 1157; *n*_test_ = 1157). The color scheme for the boxplots is arbitrary, whereas the circles on these boxplots indicate the performance for a given cross-validation repetition. The box in these boxplots shows the inter quartile range (IQR between Q1 and Q3) of the data set, the central mark shows the median and the whiskers correspond to the rest of the distribution based on the IQR [Q1–1.5*IQR, Q3+1.5*IQR]. Beyond the whiskers, data are considered outliers and represented by the diamond-shaped marker. Source data are provided as a Source Data file.

Figure 7 shows that our results (using our model and pipeline) can be reproduced by using the originally proposed model and pipeline of Peng et al. using our code as well as Schulz et al.’s code. Specifically, a highly similar performance is observed for all metrics for the SFCN Abrol^@^, SFCN Schulz^@^ and SFCN Schulz_C^*^ pipelines as compared with our DL1 Abrol^@^/DL3 Abrol^@^ pipeline (classification/regression), and this is a significant improvement over the performance of SML methods. It is easy to note from these results that the only notable change in the age regression task is in the R2 metric for SFCN Schulz^*^, which gets corrected to a higher value for the corresponding corrected pipeline (i.e., SFCN Schulz_C^*^). Some performance differences remain across the DL1 Abrol^@^/DL3 Abrol^@^, SFCN Abrol^@^, SFCN Schulz^@^, and SFCN Schulz_C^*^ pipelines, which may be explained owing to the stochastic nature of training in DL methods and parametric differences in the training pipeline. Overall, this comparative analysis enables a great insight into reproducible research confirming the similarity in performance of the considered DL models and pipelines on neuroimaging data.

## Discussion

Our results demonstrate that DL methods, if implemented and trained according to the common practices, have the potential to substantially outperform SML methods and to scale particularly well presenting a lower asymptotic complexity in relative computational time, despite being more complex in their architecture and parameterization. This observation of substantial margins in the performance graphs is consistent with the notion that the studied task associations are embedded at intricate abstract levels in the complex imaging data and therefore can benefit from the representational power of the DL methods. We further corroborated this notion by demonstrating that superior feature extraction contributed to the excellent performance of DL methods and that SML methods can perform equally well if we train them on the DL representations. Hence, we strongly recommend future work with DL methods to assess the wealth of spatiotemporal information available in the minimally preprocessed input space, as compared to working with reduced feature spaces. Note, we are not discovering something new here, the DL field is not only aware of this property of the models, but arguably they were developed with this as a primary goal^5,6,89,90^. Our analysis also suggests that the performance improvement as a function of training sample size for DL methods eventually saturates similarly to SML methods, although at a significantly higher performance mark. Although the deeper variant of the DL method tested in this work trained faster, it did not result in a significantly improved performance, therefore demanding a further probe to confirm if additional depth could further enhance the performance of the DL models. We note here that there are, nevertheless, many gateways to potentially score further performance gains apart from experimenting with even deeper variants of the tested class of DL models, for example, exploring variations in the finetuning process and testing other existing or newer DL frameworks. Indeed, it would be highly interesting to benchmark the performance and scalability bounds of an extensive battery of diverse supervised and unsupervised DL frameworks on brain imaging data.

We also illustrated that the DL embeddings from the validated models indeed capture meaningful and consistent patterns of representations of the brain that distilled continuously with more training data, spanning a comprehensible projection spectrum of age, gender and a widely used cognitive score in clinical studies. We further exemplified this by showing that DL consistently localizes discriminative brain representations, demonstrating the robustness of task-discriminative relevance estimates in multiple relevance interpretation methods. However, further substantiation of the observed robustness is recommended in future work, for example, by evaluation of the findings on a secondary data set or non-overlapping partitions of the same data set. Our observation of rational task-specific relevance distributions of discriminative biomarkers for all undertaken tasks also demands further verification, especially given the limited confirmatory knowledge on the human brain. Nonetheless, these initial results suggest the high potential of DL methods in learning specific changes in the brain that explain the differences in the analyzed groups, an attribute that holds paramount significance in important applications such as disease characterization and studying treatment effects. We presume the effectiveness of engaging DL methods in the domain of brain imaging would primarily depend on how well we can explain such vital tasks, by leveraging the existing as well as by building newer methodical interpretations for the diverse range of DL models. Eventually, the DL chalk-horses in neuroimaging would highly likely be the ones that source a healthy blend of superior representational learning and finer interpretability.

Notably, the capability of DL models to learn brain representations in an end-to-end manner does not imply that DL will necessarily perform significantly better for all learning tasks, especially not while learning from data lacking predictive signal (for example, tough problems that are barely predictable around chance levels). In addition, it may be possible to design abstract data representations (manually designed or automatically pre-engineered or a combination of these) that offer even more predictive value than made available by end-to-end trained DL models. Yet the current trends in generic computer vision clearly suggest a wide range of DL models that perform exceptionally well (better than SML methods) on tasks that can be predicted beyond chance. One may also argue that certain DL operations (for example, convolution and pooling) may decrease efficacy in specific sub-tasks (for example, learning high fractal dimension white matter bundles^91^). However, it is possible to efficiently overcome these limitations by tweaking the studied DL model or perhaps learning these patterns with other supervised/semi-supervised/unsupervised DL models^92–94^. In addition, the transfer learning efforts demonstrate that even basic segmentation models such as fully convolutional neural networks may construct representations useful for the downstream tasks^95^ and that the models without them generalize poorly. Contrarily, SML methods cannot learn such representations and are dependent on informative lower-dimensional data representations.

Large-scale data collection^96,97^, collaborative data meta-analyses^98^, and decentralized data analysis^99^ have gained enormous ground in recent years, thus providing the neuroimaging community with an unparalleled opportunity for researching big sample sizes. Yet, sample sizes for rare affected populations or isolated tasks may still be limited from tens to a few hundreds of subjects. Although DL consistently outperformed SML for larger training sample sizes in our work, DL analysis at small sample sizes (*n* = 50 and *n* = 100) did not perform worse than SML on our data. In fact, Peng et al.^29^ report a significant improvement with DL at as few as 50 subjects for this same task. Nonetheless, as we did not observe a substantial difference in the DL’s performance compared to SML at such low sample sizes, we urge the need for evaluating small sample sizes with additional caution. We also note that a future study focusing on rare low-sized data samples and isolated tasks is necessary to establish a consensus in this regard. Notably, not all tasks where DL methods perform remarkably well require many subjects, for example, segmentation. Furthermore, an active research area in the DL field is self-supervised and meta-learning, where the models are trained on larger data collections unrelated to the task at hand and then fine-tuned on small datasets. We have observed improvements in performance with this approach on training sets as small as 30 subjects^100^.

In essence, our findings highlight the presence of nonlinearities in the brain imaging data that DL frameworks can exploit to generate more discriminative encodings for characterizing the human brain. Results are in support of the potential of DL applications to brain imaging data, even with currently available data sizes; existing claims/speculations of the unlimited scalability of DL methods, however, demand further confirmation. Our findings motivate future DL work in brain imaging to focus on excelling in the discriminative power of the encodings and facilitating more precise discriminative feature localization through methodical model interpretations. Notably, the discriminative capacity of DL models is more straightforward to evaluate, but that is not the only and, arguably, the primary use that can benefit from them. A number of other applications such as segmentation and multimodal data integration directly benefit from representational powers and ease of model construction of the DL approaches. Rather than focusing on ways to show DL does not predict as well in some instances, we should be leveraging the flexibility of these models, to greatly advance in brain imaging problems that the current workhorse SML methods are not able to further push.

## Methods

### Data

The combined age and gender-based classification, gender classification, and age regression tasks in this work used sMRI images (*n* = 12,314) from unaffected subjects (i.e., those who had no diagnosed or self-reported mental illnesses based on 22,392 subjects’ sMRI data available as of 7 April 2019) from the UK Biobank repository. The sMRI data were segmented into tissue probability maps for gray matter, white matter, and cerebral spinal fluid using SPM12. The gray matter images were then warped to standard space, modulated and smoothed using a Gaussian kernel with an FWHM = 10 mm. The preprocessed gray matter volume images had a dimensionality of 121 × 145 × 121 in the voxel space, with the voxel size of 1.5 × 1.5 × 1.5 mm^3^. The MMSE regression task in this work used sMRI images (*n* = 828) from the ADNI repository (available as of 6 November 2017) and that satisfied our Alzheimer’s disease (AD) progression study criterion in our recent work^19^. The preprocessed gray matter volume images for the ADNI data had a dimensionality of 160 × 195 × 170 in the voxel space, with the voxel size of 1 × 1 × 1 mm^3^. The subjects included cognitively normal individuals (*n* = 237), individuals with MCI (*n* = 434; 245 stable MCI and 189 progressive MCI) as well as AD diagnosed individuals (*n* = 157).

The scientific study protocol of the UK Biobank is approved by the Ethics and Governance Council. Written, informed consent was obtained from all subjects participating in the UK Biobank study. The ADNI study procedures were approved by the institutional review boards of all participating centers as detailed in this document—https://adni.loni.usc.edu/wp-content/uploads/how_to_apply/ADNI_Acknowledgement_List.pdf. Written, informed consent was obtained from all subjects participating in the study according to the Declaration of Helsinki, and the study was approved by the institutional review board at each participating site.

Our choice of the sMRI modality for this project was made owing to the ease of availability of diverse and easily modifiable DL architectures for three-dimensional images. On the other hand, training on fMRI images’ four-dimensional space demands processing the additional time dimension when off-the-shelf architectures that can handle it are lacking. Nonetheless, we believe that enabling training directly from fMRI, perhaps together with sMRI, is an exciting challenge that can expand representational learning’s advantages to dynamic brain imaging. Indeed, comparing the performance of models trained directly on the minimally preprocessed fMRI data input space in an end-to-end manner with those trained on pre-engineered voxel-level or network-level fMRI features would allow for a more exhaustive benchmarking and forms an excellent topic for future research. Likewise, segmentation is not a necessity for representational learning on MRI data, and we use gray matter maps in this paper to allow a direct comparison with the previous work^28^. In fact, the DL models do exceptionally well at segmenting the data^101,102^. Also, if unsegmented data were used instead, the DL methods could appropriately identify discriminative regions in the multiple tissue structures concurrently (i.e., set up as a single problem), and possibly predict even better given the additional information accessible to them. At last, the choice of smoothing the gray matter maps, although not critical for our demonstrations, is based on results from our previous analysis on sMRI data^19^ that revealed a significant performance improvement on smoothed data (*p* < 0.05) as compared with that evaluated on non-smoothed data. This improvement is arguably owing to the marginal increase in the signal to noise ratio caused by smoothing. Moreover, the data were smoothed at the subject level (and not the group level) to retain maximal inter-individual variability (differences).

### CV procedure

A key objective of this work was to compare how classification performance on the human brain images scaled with increasing sample sizes across the different SML and DL models. To execute this, the UK Biobank data set of 12,314 subjects was stratified into three partitions: training (*n* = 10,000), validation (*n* = 1157), and test (*n* = 1157), with the training sample size varying in the range 100 to 10,000 subjects (*n* = 100, 200, 500, 1000, 2000, 5000, and 10,000) for the age and gender classification task, while using the largest training sample size partitions (*n* = 10,000) for the gender classification and age regression tasks. In a similar fashion, for the MMSE regression task, the ADNI data set of 828 subjects was stratified into training (*n* = 428), validation (*n* = 200), and test (*n* = 200) partitions. For all tested (SML and DL) models, a repeated (*n* = 20 for the age and gender tasks and *n* = 10 for the MMSE task) stratified Monte Carlo (i.e., repeated random sub-sampling) CV procedure was employed. For each repetition, the training, validation and test samples were sampled exactly once to ensure a consistent comparison by keeping them the same across the different methods. Notably, the above procedure was repeated to decrease the estimator bias and generate better estimates of the distribution of the classification performance metric. Finally, hyperparameter tuning (detailed in the following section) was employed using the training and validation folds and held-out test data samples were fed to the validated models to compute test accuracies for each CV repetition and classification approach. In summary, the primary performance comparison was employed across multiple dimensions: 20 repetitions, 7 training sample sizes, 3/none (SML/DL) dimension reduction methods, and 6/2 (SML/DL) classifiers for the classification tasks as well as three/one (SML/DL) regression models for the regression tasks.

### SML models

Six linear and nonlinear SML models (motivated by Schulz, et al.^28^) were tested for the classification tasks to estimate a diverse baseline to compare the performance of DL models. The linear approaches included LDA method, LR, and SVML models, whereas the nonlinear SML models included SVMP, SVMR, and SVMS. Three SML models, including the EN, KRR, and RF ensemble learning methods were tested in the regression tasks to estimate the baseline to compare the performance of DL models. All models were implemented with the scikit-learn python machine learning library.

### Feature extraction for SML models

Feature extraction is a crucial process to eliminate redundant features in the data to boost the performance of linear and kernel-based algorithms^3^. This step helps in reducing overfitting as lesser data dimensions imply a lesser probability of inferring decisions based on noise, and also significantly reduces the model complexity and training time of the algorithms. Hence, three dimensionality reduction methods (DRMs) were tested for feature extraction (as in Schulz et al.^28^) for all six SML models: GRP, RFE, and UFS. The GRP method projects the high-dimensional data onto a lower-dimensional subspace preserving the similarity in the data vectors using a random matrix generated using a Gaussian distribution^103,104^. Recursive feature elimination is an iterative procedure that prunes the least ranked (i.e. least significant) features in each iteration until the desired number of features is achieved^105,106^. In this analysis, we reduced 25% of the least ranked features at each step. Finally, the UFS method is based on univariate statistical tests to select the highest scoring features. We used the ANOVA F-values to return the univariate scores for feature ranking. For a consistent comparison and following the same work, the (voxel-wise) input space for each subject was reduced to a 784-dimensional subspace using each of these methods.

### Hyperparameter Validation

Hyperparameter tuning was employed for SML models through a grid parameter search with the hypopt python package. For the classification tasks, the hyperparameter grids were chosen to be consistent with the commonly evaluated parameter ranges as also employed in Schulz et al.^28^. The cost parameter (that is proportional to the inverse of the regularization strength) was tested for 10 values sampled on a logarithm scale for a range of powers of 2 from [−20, 10] for the LR and SVML models. This range of powers was shifted for all three nonlinear SVM kernel models to [−10, 20]. The other regularization parameter, gamma, was tested for 10 values sampled on a logarithm scale for a range of powers of 2 from [−25, 5] for all nonlinear SVMs. Coefficients for the poly and sigmoid kernel SVMs were tested for −1, 0, and 1 values. To ensure computational tractability, the maximum number of iterations was set to 10,000 for all four SVM classification models. The number of CPU threads to be used for the grid search class in the hypopt package was set to 8, and this parameter was kept the same in the DL training routines to allow a consistent comparison in the empirical asymptotic relative time complexity.

Similarly, several hyperparameters were tuned for all SML regression methods used in the age and MMSE regression tasks. For the KRR method, the hyperparameter grids were spanned for the kernel mapping function (linear/radial-basis-function/polynomial/sigmoidal), regularization strength (alpha in the [1e -3, 1e -2, 1e -1, 1] range) and the gamma parameter in kernel mapping functions (20 values sampled on a logarithm scale for a range of powers of 10 from [−20, 10]). For the RF method, the number of trees in the forest (10 values sampled uniformly in the [100, 200] range), maximum number of features considered at each split (chosen as square root or logarithm to the base 2 of the number of features), the minimum number of samples required to split an internal node (2, 5 or 10), the minimum number of samples required to be at a leaf node (1, 2, or 4) and the bootstrap flag (on/off) were tuned as hyperparameters. At l, for the EN regression method, the alpha tuning parameter multiplied to the penalty terms (in the [1e -1, 1e -2, 1e -3, 1e -4, 1e -5, 1e -6] range) and convex combination penalty parameter (10 values sampled uniformly in the [0,1] range) were tuned as hyperparameters.

### DL models

Two 3D-CNN variants of the AlexNet architecture^44^ were implemented on the open-source PyTorch GPU framework to establish a performance baseline for the DL models in the combined age and gender classification task. The first variant (DL1) was configured with five convolutional layers with a variable number of channels in each of the convolutional layers (64C-128C-192C-192C-128C). In contrast, the second variant (DL2) was a deeper version with six convolutional layers and an increased number of channels in the later layers (64C-128C-192C-384C-256C-256C). Only the DL1 variant was used for the gender classification task. For both regression tasks, the DL1 variant was adapted to a deep vanilla regressor (DL3) by reducing the output nodes in the final fully connect layer to one.

### DL training

Training and testing routines for the DL architectures were implemented on an NVIDIA CUDA parallel computing platform (accessing 2 Intel(R) Xeon(R) Gold 6230 CPU @ 2.10 GHz nodes on the TReNDS slurm-managed cluster each with 4 NVIDIA Tesla V100 SXM2 32 GB GPUs) using GPU accelerated NVIDIA CUDA toolkit (cudatoolkit), CUDA Deep Neural Network (cudnn) and PyTorch tensor libraries. The Adam algorithm^107^ (as implemented in the torch.optim package) for first-order gradient-based optimization of stochastic objective functions was preferred for its computational efficiency, low memory requirements, and suitability for tasks with high-dimensional parameter spaces. Our custom code also uses nipy, scipy, numpy, nibabel, and pandas packages for basic image processing and read-write operations. All package versions used in our conda environment management system are listed in the Reporting Summary.

For the DL1 method, a batch size of 32 and learning rate parameter of 10^−5^ was validated in the hyperparameter tuning stage from an initial grid parameter search (batch size: [2, 4, 8, 16, 32, 64] and learning rate: [1e -1, 1e -2, 1e -3, 1e -4, 1e -5, 1e -6] on a randomly chosen largest training sample size CV fold. These parameters were retained for the second DL architecture due to the high similarity in the architectures. For the regression tasks, the learning rate was tuned in a similar range for the DL3 method for the regression tasks. For these tasks, a batch size of 32 and learning rate parameter of 10^−5^ was validated in the hyperparameter tuning stage. For all four tasks, a learning rate scheduler callback was employed to reduce the learning rate by a factor of 0.5 on plateauing of the validation accuracy metric. Early stopping with a patience level of 20 (40) epochs was implemented on the validation accuracy metric to reduce overfitting and achieve lower generalization error in the testing phase for the classification (regression) tasks. The cross-entropy loss function was used for the classification tasks, whereas the MSE loss was used for the regression tasks. Similar to the SML models, a maximum number of 8 CPU threads were allocated to each of the DL runs for a consistent comparison in the time complexity analysis.

For the comparative analysis in this work, we compared the following pipelines on the exact same data partitions as in all previous analyses. We superscript the use of our custom trainer with an “ ^@^” symbol in the name of the pipeline and refer to the use of PyTorch lightning trainer with a “ ^*^” symbol.

1. Our DL models, our training pipeline and our custom training code (DL Abrol^@^).
2. SFCN DL model, our training pipeline and our custom training code (SFCN Abrol^@^).
3. SFCN DL model, Schulz et al. training pipeline and our custom training code (SFCN Schulz^@^).
4. SFCN DL model, Schulz et al. training pipeline and computer code (SFCN Schulz^*^).
5. SFCN DL model, Schulz et al. training pipeline and corrected computer code (SFCN Schulz_C^*^).

The training pipeline in Schulz et al. implemented an SGD optimizer with an initial learning rate of and an L2 weight decay parameter of 0.001, a learning rate step scheduler to reduce the learning rate by a factor of 0.3 every 30 epochs, and used the best validation model (i.e., at the epoch with minimum validation MAE for regression and maximum validation accuracy for classification) from 150 epochs for testing. These settings were also replicated in our code (SFCN Schulz^@^ pipeline).

In summary, this work used several standard checks for appropriately training the DL models—(1) a rigorous CV procedure, (2) a learning-rate scheduler, (3) fully automated early stopping criterion, (4) batch normalization, (5) L2 weight decay in optimizer function, and (6) dropout layers. These steps are routine in modern DL practices for combatting overfitting and thus, control for the bias-variance tradeoff frequently discussed in the field. In addition, we note that bias-variance tradeoff gains a complex interpretation in the case of overparametrized DL models as recently noted by Belkin et al.^108^. We also note that a more rigorous hyperparameter validation for the used DL methods and steps such as controlling for the number of DL model parameters, by studying space complexity could be useful for further reduction of generalization errors. These additional steps could be crucial in solving even more complex problems such as predicting cognitive scores, etc. However, hyperparameter validation in DL remains a challenge. It is itself a vast emerging field since basic random-search or grid search methods are inadequate for DL investigations owing to increased computational demand of large powerful models, whereas other proposed approaches such as Bayesian reasoning^109,110^ are not fully optimized and may suffer from the local minima problem. As such, we put DL models at a disadvantage here extensively tuning hyperparameters of SML methods in broad ranges for all four learning tasks. Meanwhile, for hyperparameter search of the DL models we employ a narrow grid search on the most important parameters such as learning rate and batch size, using defaults for the less significant ones.

### Reporting summary

Further information on research design is available in the Nature Research Reporting Summary linked to this article.

## Supplementary information

Description of Additional Supplementary Files

Supplementary Video 1

Reporting Summary

## Data Availability

The MRI data that support the findings of this work are available to researchers via the UK Biobank data access procedure described at https://www.ukbiobank.ac.uk/enable-your-research and via the Alzheimer’s Disease Neuroimaging Initiative (ADNI) data access procedure described at http://adni.loni.usc.edu/data-samples/access-data/. The UK Biobank MRI data were obtained under data application number 34175 and the ADNI preprocessed MRI data were obtained under account ID aabrol@mrn.org for a previous study (10.1016/j.jneumeth.2020.108701). Source data are provided with this paper.

## Source data

Source Data

## Code availability

The code developed for this work is available at Github: 10.5281/zenodo.4309677.
