# Supplementary material for: Deep learning encodes robust discriminative neuroimaging representations to outperform standard machine learning
Source: Nat Commun. 2021 Jan 13;12:353. doi: 10.1038/s41467-020-20655-6 (PMC7806588; doi:10.1038/s41467-020-20655-6)
Supplement: Supplementary file 1 — Description of Additional Supplementary Files [file 41467_2020_20655_MOESM1_ESM.pdf]

File Name: Supplementary Video 1
Description: Visualization of Task-Specific Distributions of Discriminative Biomarkers. The rationality of the DL model decisions was verified by examining the peak activations in the task-specific aggregate saliency maps. This animation loops over the different axial slices of the aggregate saliency maps for each of the undertaken classification/regression tasks.
