# Supplementary figures and images for: Deep learning encodes robust discriminative neuroimaging representations to outperform standard machine learning
Source: Nat Commun. 2021 Jan 13;12:353. doi: 10.1038/s41467-020-20655-6 (PMC7806588; doi:10.1038/s41467-020-20655-6)

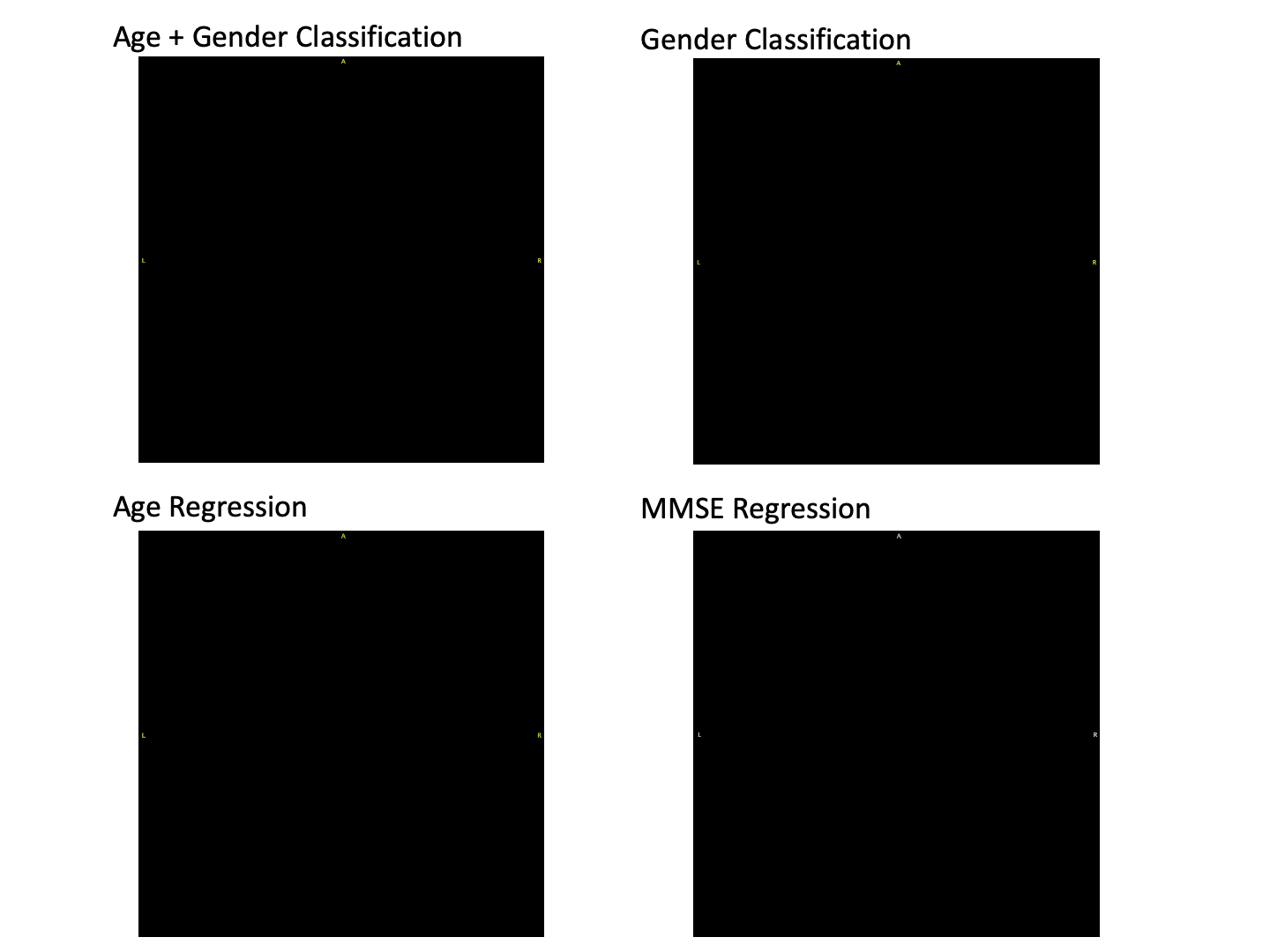

Supplement: Supplementary file 2 — Supplementary Video 1 [file 41467_2020_20655_MOESM2_ESM.gif]
