# Supplementary material for: Deep learning encodes robust discriminative neuroimaging representations to outperform standard machine learning
Source: Nat Commun. 2021 Jan 13;12:353. doi: 10.1038/s41467-020-20655-6 (PMC7806588; doi:10.1038/s41467-020-20655-6)
Supplement: Supplementary file 3 — Reporting Summary [file 41467_2020_20655_MOESM3_ESM.pdf]

nature research

Corresponding author(s): Anees Abrol

Last updated by author(s): 12/04/2020

Reporting Summary

Nature Research wishes to improve the reproducibility of the work that we publish. This form provides structure for consistency and transparency in reporting. For further information on Nature Research policies, see our Editorial Policies and the Editorial Policy Checklist.

Statistics

For all statistical analyses, confirm that the following items are present in the figure legend, table legend, main text, or Methods section.

n/a	Confirmed	
☐	☒	The exact sample size (n) for each experimental group/condition, given as a discrete number and unit of measurement
☐	☒	A statement on whether measurements were taken from distinct samples or whether the same sample was measured repeatedly
☐	☒	The statistical test(s) used AND whether they are one- or two-sided<br>*Only common tests should be described solely by name; describe more complex techniques in the Methods section.*
☐	☒	A description of all covariates tested
☐	☒	A description of any assumptions or corrections, such as tests of normality and adjustment for multiple comparisons
☐	☒	A full description of the statistical parameters including central tendency (e.g. means) or other basic estimates (e.g. regression coefficient) AND variation (e.g. standard deviation) or associated estimates of uncertainty (e.g. confidence intervals)
☐	☒	For null hypothesis testing, the test statistic (e.g. F, t, r) with confidence intervals, effect sizes, degrees of freedom and P value noted<br>*Give P values as exact values whenever suitable.*
☒	☐	For Bayesian analysis, information on the choice of priors and Markov chain Monte Carlo settings
☒	☐	For hierarchical and complex designs, identification of the appropriate level for tests and full reporting of outcomes
☐	☒	Estimates of effect sizes (e.g. Cohen's d, Pearson's r), indicating how they were calculated

Our web collection on statistics for biologists contains articles on many of the points above.

Software and code

Policy information about availability of computer code

Data collection	No software was used for data collection as data was obtained from public datasets.

Data analysis

The custom code used in this article is made available at https://github.com/aabrol/SMLvsDL/. The following packages are used:

conda (version 4.8.3)
cudatoolkit (version 10.0.13)
cudnn (version 7.6.5)
hypopt (version 1.0.9)
nipy (version 0.4.1)
numpy (version 1.17.2)
nibabel (version 2.5.0)
pandas (version 0.25.1)
python (version 3.7.4)
pytorch (version 1.2.0)
scikit-learn (version 0.21.3)
scipy (version 1.2.0)
slurm (version 19.05.0)
torchvision (version 0.4.0)
pytorch-lightning (version 0.10.0)

The Adam optimization algorithm as implemented in the torch.optim package was used.

For manuscripts utilizing custom algorithms or software that are central to the research but not yet described in published literature, software must be made available to editors and reviewers. We strongly encourage code deposition in a community repository (e.g. GitHub). See the Nature Research guidelines for submitting code & software for further information.

Data

Policy information about availability of data

All manuscripts must include a data availability statement. This statement should provide the following information, where applicable:

- Accession codes, unique identifiers, or web links for publicly available datasets
- A list of figures that have associated raw data
- A description of any restrictions on data availability

The structural MRI data used in this work are available to researchers via the UK Biobank data access procedure described at https://www.ukbiobank.ac.uk/enable-your-research and via the Alzheimer's Disease Neuroimaging Initiative (ADNI) data access procedure described at http://adni.loni.usc.edu/data-samples/access-data/. The UK Biobank MRI data were obtained under data application number 34175 and the ADNI preprocessed MRI data were obtained under account ID aabrol@gsu.edu for a previous study (doi.org/10.1016/j.jneumeth.2020.108701). Source data for the figures are provided as a Source Data file.

Field-specific reporting

Please select the one below that is the best fit for your research. If you are not sure, read the appropriate sections before making your selection.

- [x] Life sciences
- [ ] Behavioural & social sciences
- [ ] Ecological, evolutionary & environmental sciences

For a reference copy of the document with all sections, see nature.com/documents/nr-reporting-summary-flat.pdf

Life sciences study design

All studies must disclose on these points even when the disclosure is negative.

Sample size

This study did not require sample size estimation by power analysis. All available UK Biobank and ADNI data that passed quality control procedures as described in data exclusions were retained for this study.

The combined age and gender-based classification, gender classification and age regression tasks in this work used sMRI images (n = 12,314) from unaffected subjects (i.e. those who had no diagnosed or self-reported mental illnesses based on 22,392 subjects' sMRI data available as of April 7, 2019) from the UK Biobank repository.

The MMSE regression task in this work used sMRI images (n = 828) from the ADNI repository (available as of November 6, 2017) and that satisfied our Alzheimer's disease (AD) progression study criterion in our recent work (Abrol et al. 2020; https://doi.org/10.1016/j.jneumeth.2020.108701). The latter was used as a validation dataset comprising data from cognitively normal individuals and individuals diagnosed with mild cognitive impairments or Alzheimer's disease.

Data exclusions

Data was excluded through predetermined quality control procedures as detailed next.

UK Biobank: A predetermined study objective was to focus on learning age and gender based learning tasks on the unaffected population to reduce heterogeneity in the multi-group UK Biobank data. Therefore, UK Biobank subjects who had any diagnosed or self-reported mental illness based on 22,392 subjects' sMRI data (available as of April 7, 2019) were excluded from this study.

ADNI: Another objective of our study was to verify if deep learning models can learn any crucial tasks beyond age and gender, for which Mini-

	Mental State Examination was studied. This was done by using the preprocessed ADNI data from our previous deep learning study (Abrol et al. 2020; https://doi.org/10.1016/j.jneumeth.2020.108701). In that study, subjects that did not pass specific class selection criterion and the image preprocessing pipeline quality checks were excluded from the entire list of ADNI subjects data (ADNI 1/2/GO/3 available as of November 6, 2017). Specifically, healthy aging controls that had any conversions in a minimum of 3 years of follow-up (from their baseline scans), individuals diagnosed as MCI with multiple conversions in a three year follow-up and AD subjects showing reversions in a minimum of 2 years of follow-up were excluded. This exclusion was essential to eliminate confounding factors due to the unresolved diagnosis and thus allow better understanding progression to Alzheimer's disease in that study. A total number of 830 subjects passed this criterion with further elimination of only two subjects that failed the image preprocessing pipeline quality analysis thus resulting in an overall sample size of 828 subjects for this dataset.
Replication	A rigorous stratified Monte Carlo (i.e. repeated random sub-sampling) cross-validation procedure was used (n = 20) for all undertaken tasks in this study to ensure reliability of the evaluated performance metrics. The exact same train/validation/test partitions were used across all methods and the performance of all methods was assessed on held-out (unseen) data. All experiments could be successfully validated.
Randomization	Randomization was not performed and is not applicable to our study. We did not collect the MRI data but analyzed public data, and we do not study treatment effects.
Blinding	Blinding was not performed and is not applicable to our study for the exact same reasons. We did not collect the MRI data, but analyzed public data, and we do not study treatment effects.

Reporting for specific materials, systems and methods

We require information from authors about some types of materials, experimental systems and methods used in many studies. Here, indicate whether each material, system or method listed is relevant to your study. If you are not sure if a list item applies to your research, read the appropriate section before selecting a response.

Materials & experimental systems

n/a	Involved in the study
☒	☐ Antibodies
☒	☐ Eukaryotic cell lines
☒	☐ Palaeontology and archaeology
☒	☐ Animals and other organisms
☐	☒ Human research participants
☒	☐ Clinical data
☒	☐ Dual use research of concern

Methods

n/a	Involved in the study
☒	☐ ChIP-seq
☒	☐ Flow cytometry
☐	☒ MRI-based neuroimaging

Human research participants

Policy information about studies involving human research participants

Population characteristics	12,314 Unaffected UK Biobank Subjects: 6205 Females, 6109 Males ; Age: range = 45-80 years, mean = 62.6 years , standard deviation = 7.4 years<br><br>828 ADNI Subjects (237 Cognitively Normal, 245 Stable MCI, 189 Progressive MCI and 157 AD); 385 Females, 443 Males ; Age: range = 55-90 years, mean = 73.4 years , standard deviation = 6.9 years
Recruitment	No recruitment bias could be identified. Recruitment details may be found at https://www.ukbiobank.ac.uk/about-biobank-uk/ for UK Biobank and http://adni.loni.usc.edu/about/ for ADNI.
Ethics oversight	The scientific study protocol of the UK Biobank is approved by the Ethics and Governance Council. Written, informed consent was obtained from all subjects participating in the UK Biobank study. The ADNI study procedures were approved by the institutional review boards of all participating centers as detailed in this document - https://adni.loni.usc.edu/wp-content/uploads/how_to_apply/ADNI_Acknowledgement_List.pdf. Written, informed consent was obtained from all subjects participating in the study according to the Declaration of Helsinki, and the study was approved by the institutional review board at each participating site.

Note that full information on the approval of the study protocol must also be provided in the manuscript.

Magnetic resonance imaging

Experimental design

Design type	Structural MRI (T1); no task was performed.
Design specifications	MRI data preprocessing details are included in the "Data" sub-section of our "Methods" section.
Behavioral performance measures	Behavioral performance measures are not used in this study.

Acquisition

Imaging type(s)	Structural MRI
Field strength	3T
Sequence & imaging parameters	UK Biobank: Resolution: 1x1x1 mm; Field-of-view: 208x256x256 matrix; TR = 2000; TI = 880<br>ADNI 3: Resolution: 1x1x1mm; Field of View: 208x240x256mm; TR=2300; TI=900
Area of acquisition	Whole-brain

Diffusion MRI ☐ Used ☒ Not used

Preprocessing

Preprocessing software	SPM12 and in-house Matlab scripts
Normalization	Non-linear
Normalization template	MNI
Noise and artifact removal	Poorly registered scans were excluded from the analysis. See data exclusions sections on this page.
Volume censoring	No

Statistical modeling & inference

Model type and settings	Multivariate standard machine learning and deep learning classification and regression models were used for processing the structural images.
Effect(s) tested	MRI correlates of age, gender and MMSE clinical score were explored.

Specify type of analysis: ☒ Whole brain ☐ ROI-based ☐ Both

Statistic type for inference (See Eklund et al. 2016)	Whole-brain
Correction	No correction was needed as effects were estimated for the whole-brain and not at the voxel level. Further, no multiple comparisons were made.

Models & analysis

n/a	Involved in the study
☒	☐ Functional and/or effective connectivity
☒	☐ Graph analysis
☐	☒ Multivariate modeling or predictive analysis

Multivariate modeling and predictive analysis	This study used three dimension reduction methods, six standard machine learning and two deep learning classification models, as well as three standard machine learning and one deep learning regression models. Classification accuracy was evaluated for the classification tasks, whereas mean absolute error and Pearson's correlation (between the observed and true values) metrics were evaluated for the regression tasks.
